# Determining factors for Cannabis use among Moroccans Schizophrenic Patients: A cross sectional study

**Published:** 2020

**Authors:** Sara Bouri, Hanane Hanane, Khadija El Ayoubi Idrissi, Mohamed Amine Berraho, Abdelfattah Abdellaoui, Lyoussi Badiaa, Ismail Rammouz, Sanae Achour

**Affiliations:** 1 *Department of Biology, Sidi Mohamed Ben Abdellah University, Laboratory of Physiology-Pharmacology & environmental Health, Faculty of Science Fez, BP 1796 Fez-Atlas 30003, Morocco*; 2 *Department of Toxicology, University Hospital Hassan II Fez, BP 1893, road of Sidi Hrazem Fez 30000, Morocco*; 3 *Department of Psychiatry, University Hospital Hassan II Fez,BP 1893, road of Sidi Hrazem Fez 30000, Morocco*; 4 *Department of Epidemiology Public Health Pathway, Faculty of Medicine Fez, BP 1893, road of Sidi Hrazem Fez 30000, Morocco*

**Keywords:** Cannabis, Schizophrenia, Toxicological analysis, Prevalence, Comorbidity

## Abstract

**Objective:**

Cannabis use is considered a major clinical problem associated with a poorer outcome in patients with schizophrenia. The objective of the present study was to assess the prevalence of cannabis us among patients with schizophrenia. The assessment consists in comparing some factors related to substance use in a population of schizophrenic patients between cannabis users and non-

**Materials and Methods:**

Four hundred and three participants who were examined prospectively during their hospitalization answered the PANNS scale of schizophrenia, GAF, BIS-10, CDSS, and MARS. The consumption of cannabis was investigated using urine toxicological analysis. Sociodemographic, clinical and therapeutic data were also recorded.

**Results:**

The prevalence of cannabis use among schizophrenic inpatients was 49%. Patients with cannabis use were younger (31.7 vs 34.9 years old, p<0.001), more often male (52 vs 20% female, p<0.001), and they presented more often a history of imprisonment (68.8% vs 31.2%, p<0.001). Patients who were users of cannabis had a lower age at onset of the disease than non-users (23.6 vs 24.8 years, p=0.029), and more often with poor medication adherence (p=0.001). Logistic regression revealed that factors associated with cannabis use among schizophrenics were the age, gender, history of imprisonment and poor medication adherence.

**Conclusion:**

The study showed that a high prevalence of cannabis use among patients with schizophrenia which was associated with negative overall outcomes. Determining comorbid substance use disorders among schizophrenic patients is crucial as it may contribute to establish a better therapeutic strategy.

## Interpretation

Cannabis is considered the most widely used illicit substance around the world (Degenhardt et al., 2013 ▶). The use of this substance could significantly increase the risk ofdevelopment of a psychotic disorder, specifically among people who used it at an early age (Large et al., 2011 ▶; Donoghue et al., 2014; Helle et al., 2016), usually used high-potency cannabis (Di Forti et al., 2014 ▶; Marconi et al., 2016 ▶) and have a high genetic vulnerability for psychosis (Di Forti et al., 2012 ▶; Caspi et al., 2005 ▶; Mustonen et al., 2018 ▶). People with schizophrenia are more likely to use cannabis than the general population (Koskinen et al., 2010 ▶). The results from ameta-analysis of 35 studies from 16 countries revealed a median lifetime rate of cannabis use disorders of 27.1% compared with the general population (8%) (Koskinen et al., 2010 ▶). The use of cannabis is mostly associated with treatment-resistant forms of schizophrenia (Foti et al., 2010 ▶),severe positive symptoms (Foti et al., 2010 ▶; Large et al., 2014 ▶; Seddon et al., 2016 ▶), poor engagement with services and increased rates of relapse and hospitalization (Volkow, 2009 ▶;Schoeler et al., 2016 ▶), higher rate of violence (Dugré et al., 2017 ▶), and poor medication adherence (Foglia et al., 2017 ▶).Cannabis use could be a form of self-medication in order to reduce symptoms or side effects of the antipsychotic medications (Van Dij et al., 2012 ▶).

Previous epidemiological studies have reported high rates of cannabis use and cannabis use disorders among people with mental illnesses. Population-based data collected in the early 1980s by the Epidemiologic Catchment Area (ECA) study, revealed that around 50% of individuals with DSM-III psychiatric disorders meet criteria for lifetime diagnosis of cannabis use disorders (Reiger et al., 1990 ▶). In Morocco, a study carried out among 108 patients with schizophrenia, found that cannabis is the most commonly used illicit substance by patients after tobacco, with a life prevalence rate of 60% (El ghazouani et al., 2015 ▶). Most of the studies rely mainly on the patients’ self-reports regardless of the problems related to the under-report of their drug use (Carey et al., 2003 ▶; Carey and Correia, 1998 ▶; Kilpatrick et al., 2000 ▶) and there has been little evaluation of the reliability of substance use reported for patients with schizophrenia or other serious mental illnesses (Møller and Linaker, 2010 ▶). The use of biological methods tends to improve the detection of substance use over self-report assessment (de Beaurepaire et al., 2007; Bessa et al., 2010 ▶; Mieczkowski, 2010 ▶). Therefore, determining comorbid substance use disorders among patients with schizophrenia is crucial as it may contribute to establish a better therapeutic strategy.

To date, there are no studies based on the biological assessment to confirm substance use among psychotic populations in Morocco. Most of the available studies evaluating comorbidities addictions/ schizophrenic disorders were conducted in North America and Europe, and few of them were carried out in other parts of the world, especially in cannabis producing countries such as Morocco.

The present study aimed to evaluate the prevalence of cannabis use among schizophrenic patients using biological assessments, determine the risk factors associated with this consumption, and compare the socio-demographic, clinical and therapeutic characteristics between cannabis users and non-users.

## Materials and Methods

**Participants and design**

The current study was conducted in the Psychiatric Hospital Center Ibn Hassan at Fez, Morocco in collaboration with the toxicology laboratory of CHU Hassan II Fez, over a period of 14 months between May 2013 and July 2014.

The inclusion criteria for this study were: the patients must be hospitalized, with ages from 18 and 65 years, accept to participate by giving an informed consent, meet the DSM-IV-R criteria for schizophrenia, and be recorded as schizophrenics 2 years prior the beginning of the study. Patients that were excluded from the study were: non-consenting and non-cooperative patients, patients with neurological disorders and/or psychiatric comorbidity other than depression, and/or patients who were hospitalized two or more times during the period of the study. Based on the aforementioned criteria, from a total number of 512 participants (high number of patients was due to the fact that that the Psychiatric Hospital Center Ibn Hassan at fez is the only Psychiatric hospital that covers the needs of a large region), 403 patients fulfilled the criteria of the inclusion and 109 schizophrenic patients were excluded because they met one of the exclusion criteria.

The participants who met the criteria of the study were interviewed by a qualified psychiatrist or a staff member. The data were collected through a special survey document completed from: the interview with the patients, collateral information, family members, and by checking their medical records. The participants provided their informed consent after fully understanding the study. Then, they were assessed after improvement of the acute schizophrenia symptoms, and just before their discharge from the hospital.

Sociodemographic data included gender, age, marital status, living situation, employment and socioeconomic-status. These variables were coded dichotomously while education was measured categorically (never schooled, primary, and secondary). Clinical data included age of onset of schizophrenia, number of hospitalizations and the type of schizophrenia, while therapeutic data contained only antipsychotic treatment. A list of cannabis use was provided to determine the prevalence and the quantity of cannabis use, and a urinary drug screening was performed for all the participants. After urine analysis, participants underwent baseline assessment.

**Instruments**

*Cannabis Use*

The rate of use (frequency of cannabis use) and average consumption (in grams) were assessed for a period of 3 months before the interview. The use of cannabis was evaluated using: self-report (patients’ self-reports), and collateral report (family members reporting the cannabis use of the patients during the previous months). Schizophrenic patients were also subject to urine toxicological analysis for cannabis (Δ9-tetrahydrocannabinol). This analysis was carried out on a fresh urine sample that was collected from patients one day after hospitalization. Drug urine analysis was performed using both a rapid multiple immunoassay urine drug test cassette with a detection threshold 50 ng/l and ELISA test (OLYMPUS analyzer) with a threshold of 25 ng/l.

**Clinical assessments**

All the schizophrenic patients were assessed by a trained psychiatrist using the following psychometric scales to assess the psychiatric disorders: The Positive and Negative Syndrome Scales (PANSS) (Kay et al., 1987 ▶) were used to assess positive, negative, and general psychopathology symptoms. Global functioning was assessed using the Global Assessment of Functioning Scale (GAF) (American Psychiatric Association, 2003 ▶). Impulsivity was assessed using the Barratt Impulsiveness Scale (BIS-10) (Patton et al., 1995 ▶). Depression was assessed using the Calgary Depression Scale for Schizophrenia (CDSS) (Ddington et al., 1993 ▶), and medication adherence assessment was done based on Medication Adherence Rating Scale (MARS) (Thompson et al., 2000 ▶).

**Statistical analysis**

Data were analyzed using SPSS software version 21 (Statistical Package for Social Sciences). Descriptive statistical analyses were conducted for patients’ demographic and clinic information. The chi-square test was used to compare the socio-demographic, therapeutic and clinical characteristics of patient’s users and non-users of cannabis. For the categorical variables, the t test was used to compare the means of quantitative data. Multivariate analysis was performed to identify factors associated with cannabis use*. *Odds ratio (OR) and 95% confidence interval (95% CI) were calculated*. *A p value of < 0.05 was accepted as statistically significant.

## Results

**Participants**

From the 403 patients who have enrolled, 90% were men. The mean age was 33.3 years (±9.1 years). Most patients were single (76.2%), and unemployed (72.7%) and had a low socio-economic status (88.1%). A percentage of patients (45.3%) had a secondary level of education. Most of the patients included in this study had the diagnosis of paranoid schizophrenia (62%), followed by disorganized schizophrenia (19.1%). The average age of onset was 24.24±5.7 years and the average number of hospitalizations was 4.5±4.7. The average length of inpatient stay was 13.44±7.20 days. Therapeutically, the majority of hospitalized schizophrenic patients had received typical antipsychotic monotherapy (89.3%).

**Cannabis use**

From 403 patients who were screened in this study, 198 (49%) were positive for THC. The average age of onset of cannabis use, was 18.85±4.65 with an age range of10 to 39 years. As expected, cannabis use was much more frequent among males (52.3 vs. 20% female). The percentage of patients who used cannabis regularly was 39.4%. and 20.2% of them had used it occasionally.

**Comparison of cannabis users and non-users**

Table 1shows the comparison of users and non-users of cannabis, based on the socio-demographic, clinical and treatment characteristics. Regarding socio-demographic characteristics, there was a significant difference in terms of the age, gender, marital status, and history of imprisonment between the two groups. Patients with cannabis use were younger (31.7 vs 34.9 years, p<0.001), and usually male (52 vs 20% female, p<0.001), and they presented more often a history of imprisonment (68.8 vs 31.2%, p<0.001). Table 2 shows that users of cannabis had lower ages at the onset of the disease compared to the non-users (23.6 vs 24.8 years, p=0.029), and more often showed medication non-adherence (p=0.001). We did not notice any difference between the users and non-users of cannabis in terms of the type of schizophrenia, number of hospitalization, and the type of medication use.

Table 2 shows the correlation between the psychometric scales and cannabis use among schizophrenic patients. Cannabis using patients had significantly higher scores for BIS-10 (impulsiveness) total score than non-using patients (65. 8vs 63.9; p=0. 01). Besides, we found a significant correlation between medication adherence (MARS) and cannabis use among schizophrenic patients. An important significant frequency of medication adherence (76%) among cannabis non-users (76 vs 24%, p=0.009) was demonstrated. Regarding the symptoms of schizophrenia (assessed by PANSS), depression symptoms (evaluated by the CDSS), and the global functioning (GAF), the results showed no correlation between these parameters and the use of cannabis.

**Table 1:** Comparison of schizophrenic patients with and without cannabis consumption: socio-demographic characteristics

Variables	All patients (N=403) N(%)	non users (N=205) N(%)	users of cannabis (N=198) N(%)	p
Age (mean, SD)	33.35*± *9.16	34.93±9.47	31.71±8.54	0,000
**Sex**				
**Male**	363 (90.1)	173 (47.7)	190 (52.3)	0,000
**Female**	40 (9.9)	32 (80.0)	8 (20.0)	
Marital status				
**Single**	307 (76.2)	146 (47.6)	161 (52.4)	0,043
**Married**	62 (15.4)	40 (64.5)	22 (35.5)	
**Divorced**	34 (8.4)	19 (55.9)	15 (44.1)	
Children				
**No children**	340 (84.4)	167 (49.1)	173 (50.9)	0,102
**Have children**	63 (15.6)	38 (60.3)	25 (39.7)	
Living situation				
**Alone**	341 (84.6)	179 (52.5)	162 (47.5)	0,126
**With family**	62 (15.4)	26 (41.9)	36 (58.1)	
Education				
**Never schooled**	56 (13.9)	34 (60.7)	22 (39.3)	0,262
**Primary**	164 (40.8)	82(50.0)	82 (50.0)	
** Secondary**	182 (45.3)	88 (48.4)	94 (51.6)	
Occupation				
**Unemployed**	293 (72.7)	148 (50.5)	154 (49.5)	0,815
**Employed**	109 (27.2)	57 (51.8)	53 (48.2)	
Socioeconomicstatus				
**Lower**	355 (88.1)	182 (51.3)	173 (48.7)	0,760
**Middle &higher**	48 (11.9)	23 (47.9)	25 (52.1)	
Environment				
**Rural**	309 (77.4)	163 (52.8)	146 (47.2)	0,229
**Urban**	90 (22.6)	41 (45.6)	49 (54.4)	
History of imprisonment				
**No**	309 (76.6)	175(56.6)	134 (43.4)	0,000
**Yes**	93 (23.1)	29 (31.2)	64 (68.8)	

**Logistic regression**

Logistic regression (Table 3) shows that the factors that were significantly associated with the risk of cannabis use were age (OR 0.96, CI 0, 93-0, 98, p=0.001), protective risk factors included 

female sex (OR 0.32, CI 0, 14-0, 73, p=0.007), history of imprisonment (OR 2.63, CI 1, 57-4, 39, p=0.001), and medication non-adherence (OR 5.27, CI 1, 70-16, 23, p=0.001).

**Table 2:** Comparison of schizophrenic patients with and without cannabis consumption: clinical and therapeutic characteristics

Variables	non users(N=205) N (%)	users of cannabis (N=198) N (%)	p
**Type of schizophrenia**			
**Paranoid**	118(47.2)	132 (52.8)	
**Disorganized**	39 (50.6)	38 (49.4)	
**Residual**	4 (57.1)	3 (42.9)	0.189
**Undifferentiated**	12 (66.7)	6 (33.3)	
**Schizo-affective**	32 (62.7)	19 (37.3)	
**Antipsychotictreatment**			
**Typical antipsychotics**	177 (49.6)	180 (50.4)	0.102
**Atypicalantipsychotics**	27 (62.8)	16 (37.3)	
**Treatment dropout**			
** No**	21 (84.0)	4 (16.0)	0.001
** Yes**	183 (48.8)	122(51.2)	
**CDSS (depression)**			
**No**	183 (50.1)	182 (49.9)	0.363
**Yes**	22 (57.9)	16 (42.1)	
**MARS (adherence)**			
**No**	186 (49.2)	192 (50.8)	0.009
**Yes**	19 (76.0)	6 (24.0)	
**Age of onset of schizophrenia (mean±SD)**	24.84±5.98	23.60±5.34	0.029
**Duration of psychotic illness(mean±SD)**	10.14±7.70	8.10±6.44	0.004
**Number of hospitalization (mean±SD)**	4.92±5.22	4.14±4.08	0.096
**PANSS Total (mean±SD)**	59.01±11.48	59.33±10.60	0.769
**PANSS Positive (mean±SD)**	20.15±5.35	20.19±4.92	0.944
**PANSS Negative (mean±SD)**	15.23±5.35	16.10±5.63	0.128
**PANSS Psychopathology (mean±SD)**	23.61±5.73	23.04±10.60	0.292
**GAF (general functioning) (mean±SD)**	25.46±5.73	24.48±5.44	0.790
**BIS-10 (impulsivity) (mean ±SD)**	63.96±6.13	65.85±6.29	0.002

**Table 3:** Logistic regression assessing factors associated with cannabis consumption

Variable	Odd ratio adjusted	95% CI	p
Age(for 1 year)	0,96	0,93-0,98	0,001
Sex			
**Male**	1	-	0,007
**Female**	0,32	0,14-0,73	
History of imprisonment			
** No**	1	-	0,001
** Yes**	2,63	1,57-4,39	
Non-adherence			
** No**	1	-	0,004
** Yes**	5,27	1,70-16,23	

## Discussion

To the best of our knowledge, this is the first study that was conducted in Morocco to evaluate the prevalence of cannabis use among schizophrenic patients using toxicological analysis. This approach aims to confirm cannabis use based on toxicological analysis the patients’ self-reports about the cannabis use and to increase the reliability of the study. The main result of this study was that 49% of the schizophrenic patients use cannabis. This prevalence is in a good agreement with data reported by Green et al. (2005) ▶, in which they found a mean of 42.2% lifetime prevalence of cannabis use within 53 treatment samples (53 Studies included in the analyses of clinical data). This high prevalence among schizophrenic patients can be interpreted in two ways: on one hand, schizophrenia and cannabis use reinforce each other. On the other hand, cannabis use improves the well-being of the patients by managing their stress, improving their social interactions, reinforcing their control of psychotic symptoms, and reducing the side-effects of the antipsychotic treatment.

Some differences in terms of sociodemographic characteristics and clinical outcomes were found between the schizophrenic patients; users and non-users of cannabis. The multivariate analysis showed that genders, age, history of imprisonment and medication non-adherence are associated with the use of cannabis among the patients with schizophrenia. 

**Socio-demographic and clinical characteristics**

The analysis of the results showed that the patients with cannabis use are young, and predominantly male, and had a lower age at the onset of the disease (schizophrenia). These characteristics are consistent with the findings reported by the previous studies (Compton et al., 2009 ▶; Large et al., 2011 ▶; Di Forti et al., 2014 ▶). The parameter of lower age at the onset of schizophrenia could be interpreted in two different ways: either cannabis use triggers the symptoms of schizophrenia for the individuals who have a genetic predisposition of the disease; or the early onset of symptoms leads the patients to use cannabis as a self-medication to cope with their disease (Hambrecht and Hafner, 1996 ▶; Degenhardt et al., 2003 ▶). The hypothesis of self-medication has not been widely supported by the previous studies done in the regard (Henquet et al., 2005a ▶; Henquet et al., 2005b ▶)

The multivariate analysis showed that male schizophrenic patients present higher odds of cannabis use compared to female patients. So, the gender of the patient is a risk factor for male and a protective factor for female. These findings are in a good agreement with those shown by previous studies where male gender and youth (age of the patient) are risk factors for substance abuse (Cantor-Graae et al., 2001 ▶; Jimenez-Castro et al., 2010 ▶). The results of the present study also showed that the history of imprisonment is another risk factor that could increase the vulnerability to use cannabis among schizophrenic patients. These findings are consistent with those from other studies which showed that the risk of criminality among schizophrenic patients with substance use is high (Schanda et al., 2010 ▶). A longitudinal study conducted ina sample of 1353 Norwegians, aged from 13 to 27 years old, found that cannabis use was associated with drug-related offences and alcohol with violent behavior (Pedersen and Skardhamar, 2010 ▶; Norström and Pape, 2010 ▶). Further results from a meta-analysis (data from 20 different studies and a sample of 18,423 participants) concluded that the risk of violence is relatively high within individuals with psychosis (of both genders) and substance-abuse comorbidity compared with general population controls (Fazel et al., 2009 ▶). The history of imprisonment factor can be interpreted by the fact that the use of cannabis may precipitate or contextualize the use of violence among schizophrenic patients.

Medication non-adherence was found as another important risk factor related to the cannabis use among schizophrenic patients. This result is supported by the findings of a meta-analysis of 11 studies conducted by Foglia et al. (2017) ▶ in which they found that cannabis use is associated with a nearly 150% increase in the risk of non-adherence for cannabis users as compared to non-users (OR 2.46, CI 1.97–3.07, p<0.00001). Another study conducted by Miller et al. (2009) ▶ showed that cannabis use increases both the non-adherence to medication treatment for schizophrenia and the dropout of treatment. However, there are some conflicting reports regarding the correlation between cannabis use and non-adherence to medication or treatment dropout. For instance, linszen et al. (1994) ▶ showed no differences in non-adherence to medication treatment between users and non-users of cannabis.

**Cannabis consumption is not associated with higher severity of psychiatric symptoms**

Several studies reported that psychoactive substances can aggravate or precipitate the positive symptoms (delirium, and hallucination) among the majority of schizophrenic patients. However, it seems possible that they alleviate the negative symptoms, particularly anhedonia, social inhibition, or even cognitive failures (Grech et al., 2005 ▶).

This study showed no difference in symptoms of schizophrenia between the cannabis users and non-users as they have almost same levels of depression as well as positive and negative symptoms of schizophrenia. This leads to a limitation in the interpretation of the results. The absence of any deference is due to the fact that all the participants, whether they use cannabis or not, were subject to hospitalization and medical treatment. This may be the reason behind the absence of the differences in psychotic, depressive, or anxious symptoms.

**Impulsiveness and cannabis use**

Our results showed that in a group of patients with schizophrenia, the mean scores, based on the BIS scale, are higher for users compared to non-users of cannabis. The results of the present study are similar to those from previous studies evaluating impulsivity in a sample of two independent schizophrenic groups with substance abuse disorders (Dervaux et al., 2001 ▶; Gut-Fayand et al., 2001 ▶). It was not possible to determine the relationship between impulsivity and cannabis use because of the nature of the research (a cross-sectional study).

The reasons why impulsivity could be associated with substance use still need to be clarified. Several interpretation of the relationship between impulsivity and substance abuse are given in the literature. Gut-Fayand et al. (2001) ▶ suggested that high impulsivity may result in substance use as a maladaptive behavior in response to prodromal symptoms, precipitating the onset of psychosis. However, Liraud and Verdoux, (2000) ▶ concluded that the impulsivity might lead to substance abuse in a non-specific manner. Another possible interpretation, which complements the first one, was proposed by Hogarth (2011) ▶: impulsivity increases hypersensitivity to drug reinforcement which leads to high rates of drug-seeking/taking. A prospective longitudinal follow-up of a cohort of young subjects, studying the occurrence of impulsivity, sensation seeking, substance abuse, and/or schizophrenia, would be more reliable but hardly feasible.

In this study, no apparent associations between cannabis use and general functioning as well as the number of hospitalization were observed. Despite of these mixed findings, some studies found that people with psychosis who regularly use cannabis suffer from more positive symptoms, more frequent relapses and higher numbers of hospitalization (Grech et al., 2005 ▶; Schoeler et al., 2017 ▶). Cannabis use increases the risk of relapse, the number of relapses as well as their length, care intensity index at follow-up, and time until a relapse occurred (Gregg et al., 2007 ▶).

**Reasons for substance use in people with schizophrenia**

Several reasons lead patients with schizophrenia to abuse substances more specifically cannabis. Previous studies demonstrated that schizophrenic patients tend to use substance abuse (or illicit drugs) as a self-medication for tension, low mood, anxiety as well as negative symptoms (Drake, 2008 ▶; Lobban et al., 2010 ▶; Charles and Weaver, 2010 ▶). The first reason is the self-medication hypothesis that has been refuted by several studies (D'Souza et al., 2009 ▶; Dubertret et al., 2006 ▶; Potvin et al., 2006 ▶) and it found lower negative symptom score (e.g. in emotional withdrawal) among cannabis-dependent patients with schizophrenia (Larsen et al., 2006 ▶; Arndt et al., 1992 ▶). People with psychosis may use cannabis because of its benefits such as reduction of anxiety and increased sociability (Larsen et al., 2006 ▶). Another reason that leads patients to consume cannabis is the personality trait. As for non-schizophrenic patients, high levels of impulsivity and sensation seeking, play a role in promoting addictive behaviors in patients with schizophrenia (Gut-Fayand et al., 2001 ▶; Liraud and Verdoux, 2000 ▶). Also, antisocial personality traits are associated with substance use among patients with schizophrenia (Mueser et al., 1997 ▶).

## Limitation

Possible limitations of this study included the predominance of the male gender, exclusively hospital recruitment (exclusively inpatients), absence of the long-term longitudinal follow-up of patients, and the point that cannabis metabolites in urine can persist for several weeks. This may lead to false interpretations of the patients’ reports as their answers correspond to prior periods before their hospitalization. Another limitation of the present study is that the sample of patients included is not representative of the general population of the Moroccan schizophrenic patients. Exclusion of patients who are not able to interact with interviewers due to the severity of their psychiatric state, can be considered another limitation.

To our knowledge, this is the first study to confirm the prevalence of cannabis use among schizophrenic patients hospitalized using urine toxicological analysis. The findings of this study indicated that cannabis use is prevalent among hospitalized schizophrenic patients, particularly among young and male patients. This is often associated with medication non-adherence and a history of imprisonment. 

Therefore, schizophrenic patients should be sensitized to the negative effects of cannabis in order to make them not using or at least reducing their cannabis use. Hospitalization might provide a possibility to give the required information and treatment for such frequent addictive disorder. More prospective studies are still needed to explain the effects of cannabis consumption and the cannabis use disorders on schizophrenia. Finally, this study reveals the need for local epidemiological and clinical studies on substance or cannabis use disorder in schizophrenia, in order to ensure that therapeutic interventions are targeted more effectively.
